# The Effect of Propofol Anesthesia on the Pain Severity and Frequency of Migraine Attacks in Patients with Chronic Migraine Headache over a Six Month Follow Up: An Observational Study

**DOI:** 10.22037/ijpr.2021.115243.15266

**Published:** 2021

**Authors:** Maryam Vosoughian, Nima Saeedi, Mohammadreza Moshari, Shideh Dabir, Mastaneh Dahi, Soudeh Tabashi, Khadijeh Haji Naghi Tehrani, Nastaran Hajizadeh

**Affiliations:** a *Anesthesiology Research Center, Department of Anesthesiology and Critical Care, Taleghani Hospital, Shahid Beheshti University of Medical Sciences, Tehran, Iran.*; b *Department of Anesthesiology and Critical Care, Shahid Beheshti University of Medical Sciences, Tehran, Iran. *; c *Department of Neurology, Islamic Azad University, Medical Branch, Tehran, Iran. *; d *Faculty of Paramedical Sciences, Shahid Beheshti University of Medical Sciences, Tehran, Iran.*

**Keywords:** Migraine, Headache, Propofol, Pain Management, Anesthesia

## Abstract

Propofol is a short-acting intravenous anesthetic that is commonly used for induction and maintenance of anesthesia. Subanesthetic low doses of propofol has also been used to treat intractable migraine attacks in emergency wards with dramatic results. However, there is little information on the long-term efficacy of this drug in migraine headaches. The aim of this nonrandomized prospective observational study was to assess the effect of propofol anesthesia on the pain severity and frequency of migraine attacks in a 6-month follow-up period after anesthesia in patients with migraine headaches. The study was conducted on 51 known cases of migraine ranging in age from 21 to 66 years. Before anesthesia, patients completed a questionnaire including their characteristics, pain intensity of the headache using a visual analog scale, and a number of headache repetitions per month. All patients received propofol as the main anesthetic agent. At the end of anesthesia, the total amount of propofol usage was recorded. Patients were then followed up by telephone in the first, third, and sixth months after anesthesia, and the severity and frequency of the headache were recorded. Pain intensity or pain frequency significantly improved in 22 patients (43.1%), remained unchanged in 24 (47%), and worsened in 5 cases (9.8%) 6 months after anesthesia compared to before the anesthesia. In conclusion, since about half of the patients had significant improvement in the headache, propofol anesthesia may be considered as an acceptable anesthetic method in patients with migraine.

## Introduction

Migraine is a disturbing disorder that has been known to affect humankind since antiquity. Today, it is the third most common disease in the world and affects more than 10% of the population worldwide. It is characterized by moderate to a severe pulsatile painful unilateral headache that may be accompanied by vomiting, nausea, photophobia, osmophobia, and phonophobia. Women suffer from migraine more than twice than men. Migraine is also ranked among the top ten debilitating diseases and the fourth most burdensome disease in women. According to the 2012 Global Burden of Disease study, the disease has led to drug abuse, but the cause remains unclear (1-3). Propofol (2, 6 di-isopropyl phenol), a rapid and short-acting intravenous anesthetic, is a gamma-aminobutyric acid (GABA) receptor agonist which is commonly used for induction or maintenance of general anesthesia as well as sedation in diagnostic and therapeutic procedures or in intensive care patients. It is also effective in treating postoperative nausea and vomiting, refractory status epilepticus, and opioid-related pruritus. Moreover, sedative doses of propofol have resulted in significant improvement in intractable migraine attacks in the emergency wards (4-10). However, there is little information on the long-term efficacy of this drug in migraine headaches. To our knowledge, there is only one study that evaluated the effect of propofol on migraine in a one-month follow-up (11). Therefore, due to the high prevalence of migraine headaches, this study was performed to assess the long-term effectiveness of propofol anesthesia in the severity and frequency of headaches in patients with migraine over a period of 6-month after anesthesia. If this drug has lasting positive effects on the headache, it may be a good anesthetic agent in migraine patients who are a candidate for surgical and non-surgical procedures. 

## Experimental

This nonrandomized prospective observational study was conducted on 60 American Society of anesthesiologists Class *I *and *II* patients with known migraine undergoing anesthesia for various procedures, included in the study between June 2018 and December 2019 at the Taleghani Educational Hospital in Tehran. The study protocol was approved by the Research Ethics Committee of Shahid Beheshti University of Medical Sciences in Tehran, Iran (ethics code of IR.SBMU.MSP.REC.1398.169). The diagnosis of migraine was confirmed by using the International Classification of Headache Disorders, 3^rd^ edition (ICHD-3) (12). Before anesthesia, informed consent was obtained from each patient, and they filled out a questionnaire that included patients’ characteristics and the pain intensity using a visual analog pain score (VAS) and a number of repetitions of migraine attacks per month. Patients with a known allergy to propofol, hypovolemia, epilepsy, severe cardiac disease, and head and neck surgery were excluded. All patients received propofol as the main anesthetic agent and low doses of lidocaine, midazolam and fentanyl. At the end of the procedure, the total amount of propofol consumption was recorded. Patients were then followed up by telephone in the first, third, and sixth months after anesthesia and the intensity and frequency of their headache were recorded.

The data were entered into Microsoft Excel and analyzed using students *t*-test, chi- square test, and ANOVA. Considering the 10% frequency of migraines in the population and that propofol reduces its frequency by 20%, a sample size of at least 32 cases was calculated. A *p*-value of < 0.05 was considered statistically significant. 

## Results

Nine patients were excluded from the study for various reasons; three people did not answer the phone call, one person died during the follow-up, three people did not cooperate, and two phone numbers were wrong. There were 5 men (9.8%) and 46 women (90.2%) in the remaining 51 patients ranging in age from 21 to 66 years. The patients’ characteristics are shown in Table 1.

**Figure 1:**
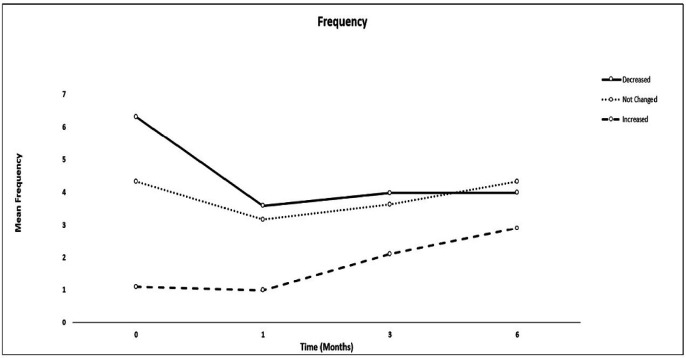
Comparisons of the mean frequency of migraine attacks between patients with decreased, unchanged and increased migraine attacks in four-time intervals

**Figure 2:**
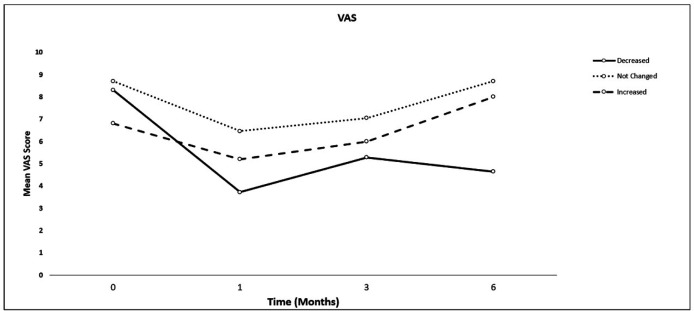
Comparisons of the mean pain scores of migraine attacks between patients with decreased, unchanged and increased migraine attacks in four-time intervals

Six months after receiving propofol anesthesia compared to the pre-anesthesia period, the number of attacks (frequency) or severity of the headache significantly improved in 22 (43.1%) patients, remained unchanged in 24 (47%), and worsened in 5 (9.8%) patients. There was no significant difference in the total dose of propofol usage and the amount of propofol given according to the patients’ weight between the patients (Table 2). The comparisons of VAS and frequency of headache are shown in Table 3. In the patients with improved headache, VAS or frequency of the migraine attacks significantly decreased in the first, third, and six months after anesthesia compared to before anesthesia (*P < *0.05), but they were similar between the three-time intervals following propofol anesthesia. In the patients with unchanged headaches, no significant difference was observed in the frequency and VAS of the headache compared to prior to anesthesia. In the 5 patients with intensified headache, VAS scores were similar in the first, third and sixth months after anesthesia compared to before anesthesia, while the frequency of migraine attacks in the third and sixth months was significantly higher than before anesthesia (*P* = 0.049). However, no significant difference was observed between preanesthesia and the first month after anesthesia (Figures 1 and 2).

## Discussion

Based on the results of our study, 6 months after propofol anesthesia, the number of migraine attacks and pain severity of the headache significantly improved in 22 patients (43.1%), did not change in 24 patients (47%), and worsened in 5 patients (9.8%) compared with pre-anesthesia period. In the patients with unchanged headaches, both pain intensity and frequency remained unchanged. The analysis of all 51 patients showed that the number and severity of migraine attacks after receiving propofol were significantly reduced at all measured time intervals. 

Despite extensive research on the pathophysiology of migraine, the mechanism of the disease is still unclear, and therefore there is a wide range of pharmacological and non-pharmacological therapeutic methods for migraine in the literature (2). The International Association of Headache introduced nonsteroidal anti-inflammatory drugs and ergotamine compounds as common treatments for mild to moderate migraines and selective 5-hydroxytryptamine (5-HT) receptor agonists such as sumatriptan for more severe cases. Intravenous metoclopramide and prochlorperazine as well as subcutaneous sumatriptan should be offered to eligible adults with acute migraine presenting to an emergency ward. Dexamethasone has also been given to these patients to prevent recurrence of the headache (7). Furthermore, low doses of propofol have been used successfully to treat refractory migrainous and nonmigranous attacks (5-11). The actual anti-migraine mechanism of propofol in treating migraine is not yet completely understood. Its main mechanism of action may be due to the inhibition of N-Methyl-D-aspartate (NMDA) receptors. Glutamate is an excitatory neurotransmitter that binds to NMDA receptors and is involved in central sensitization and migraine progression. The higher levels of glutamate have been detected in the cerebrospinal fluid of persons with migraine compared to those without migraine and have been found to be increased during migraine attacks. In animal migraine models, glutamate-induced cortical spreading depression was associated with trigeminal nerve activation and development of central sensitization. NMDA receptor antagonism is believed to prevent cortical spreading depression, a neurologic process that may potentially cause migraine and migraine aura. It has yet to be determined if propofol directly affects cortical spreading depression in humans. Propofol is also thought to inhibit calcium influx via calcium channels. 

Moreover, it can potentially prevent catecholamine-induced vasoconstriction (9, 13). The propofol efficacy may also be related to its anti-inflammatory effects through inhibition of stimuli-induced cytokines production (14). The drug has also been shown to have antioxidant properties (15). The sedative and anticonvulsant effects of propofol as well as its positive effects on postoperative nausea and vomiting are mediated by gamma-aminobutyric acid receptors (GABA) (9). 

 Available studies on the effects of propofol on chronic headaches are limited to case series (8, 10 and 16), case reports (17, 18), and very few clinical trials (19, 20). A review of several studies by Piatka *et al.* (9) showed that propofol for treating migraine in the emergency department needs sufficient experienced staff and standard monitoring that can be limiting factors for routine application of the propofol. Giampetro *et al.* (11) examined the long-term effects of propofol on migraine in a one-month follow-up. They found that subjects with chronic headaches who were exposed to propofol for endoscopy reported an improvement in their headache 30 days after the procedure. They suggested that propofol therapy plays a major role in lessening the impact of headaches on various aspects of patients’ lives, including headaches per month and pain quality. In a small case-control series study, Sheridan *et al. *(10) showed that the use of propofol in anesthetic doses might be an effective and safe drug in the treatment of pediatric migraine headaches in the emergency department. In a prospective, randomized controlled trial, Sheridan *et al. *(20) compared the efficacy of low-dose propofol with standard therapy to treat pediatric migraine. They concluded that low dose propofol was not superior to standard therapy in reducing the severity of migraine headaches and significantly shortening the median length of stay in the emergency ward; however, propofol administration was associated with significantly fewer recurrence of headache. Moshtaghion *et al*. (19) compared propofol with sumatriptan as a rescue medication in migraine in the emergency department. They found that propofol is equally suitable as sumatriptan, and it can be an ideal treatment for acute migraine in the emergency ward and hospital setting. It was also effective for the control of nausea and vomiting that are common symptoms in migraine patients. In addition to migraine headache, the efficacy of propofol in treating post-dural puncture headache (PDPH) has been reported. PDPH has a migraine-like mechanism and responds well to anti-migraine medications (21- 23). 

In our study, although the propofol dosage based on patients’ weight was not significantly different between the patients, the variety of procedures and duration of surgery might affect the results. Moreover, our study was conducted over a period of 1.5-year, and therefore the influence of environmental factors, including seasonal and psychological and hormonal impact in women, cannot be ignored. These limitations are related to the nature of the observational method of our study. Therefore, it is suggested that future controlled clinical trials investigating the effect of propofol on migraine be designed by unifying the type of operations and environmental factors. We also recommend future clinical trials to investigate the impact of propofol anesthesia *vs.* anesthesia without the usage of propofol on migraine intensity.

We performed this descriptive study hoping that it will be an initial step in finding a suitable anesthesia method to reduce postoperative migraine headaches in patients suffering from this distressing disorder. 

**Table 1:** Patients’ characteristics. Data are presented as mean ± SD and numbers

**Variables**	**Values**
Age (year)	42.96 ± 10.44 (21-66)
Weight (kg)	69.04 ± 10.94 (42-99)
Gender (Female/Male)	5/46
Surgery length (min)	38.52 ± 39.95
Surgery type	
GI endoscopy	23
Hysteroscopy	13
Orthopedic	6
Egg collection	5
Gynecologic laparoscopy	2
Mastectomy	1
Cholecystectomy	1

**Table 2:** Comparison of intravenous propofol usage between patients with decreased, unchanged and increased migraine attacks. Values are presented as mean ± SD

**Variables**	**Decreased (n = 22)**	**Unchanged (n = 24)**	**Increased (n = 5)**	***P-*** **value**
Intraoperative propofol (mg)	242.27 ± 77.58	200 ± 92.45	236 ± 94.76	0.245
Intraoperative propofol (mg/kg)	3.39 ± 1.16	2.95 ± 1.29	3.87 ± 0.96	0.229

There was no significant difference (*P > *0.05).

**Table 3:** Comparison of pain scores (VAS) and frequency of headaches between patients with decreased, unchanged and increased migraine attacks in four-time intervals. Values are presented as mean ± SD

**Time**	**Migraine headache**	**Decreased(n = 22)**	**Unchanged(n = 24)**	**Increased (n = 5)**	***P-*** **value**
Before anesthesia	VAS	8.32 ± 1.46	8.71 ± 1.23	6.80 ± 2.17	.033*
	Frequency	6.32 ± 6.66	4.33 ± 6.34	1.100 ± 0.22	.211
1 month after anesthesia	VAS	3.73 ± 3.91	6.46 ± 4.04	5.20 ± 3.56	.074
	Frequency	3.59 ± 6.48	3.17 ± 4.92	1.00 ± 0.61	.636
3 months after anesthesia	VAS	5.27 ± 2.68	7.04 ± 3.22	6.00 ± 3.67	.153
	Frequency	3.98 ± 5.91	3.63 ± 5.16	2.10 ± 1.82	.777
6 months after anesthesia	VAS	4.64 ± 2.92	8.71 ± 1.23	8.00 ± 1.87	.000^*^
	Frequency	3.99 ± 6.42	4.33 ± 6.34	2.90 ± 1.17	.892

^*^There were significant differences between the groups. VAS: visual analog scale.

## Conclusion

Because of significant improvement in the headache in about half of the patients, propofol anesthesia may be considered an acceptable anesthetic method in patients with migraine. It may be effective in long-term decrease of frequency and pain severity of the headache.

## Conflict of interest

 There is no conflict of interest.

## Funding/Support

 There is no funding support. 

## Author contributions

 All the authors contributed substantially and equally to the conception or design of the work, data analysis or interpretation, and drafting the work or revising. 
